# Investigation of Potential Inhibitors of N‐Myristoyltransferase in *Leishmania amazonensis*: A Computational and Experimental Study

**DOI:** 10.1111/cbdd.70170

**Published:** 2025-09-08

**Authors:** Mariana Sant’Anna Pereira Nicolau, Millena Almeida Resende, Cintia de Campos Chaves, Renata Santos Rodrigues, Veridiana de Melo Rodrigues, Nilson Nicolau‐Junior, Kelly Aparecida Geraldo Yoneyama

**Affiliations:** ^1^ Laboratory of Biochemistry and Animal Toxins, Institute of Biotechnology Federal University of Uberlandia Uberlandia MG Brazil; ^2^ Laboratory of Molecular Modeling, Institute of Biotechnology Federal University of Uberlandia Uberlandia MG Brazil

**Keywords:** computer‐aided drug design, drug discovery, enzyme inhibition, *Leishmania amazonensis*, Leishmaniasis, N‐myristoyltransferase (NMT)

## Abstract

Leishmaniasis, a disease caused by *Leishmania* parasites, poses a significant health threat globally, particularly in Latin America and Brazil. *Leishmania amazonensis* is an important species because it is associated with both cutaneous leishmaniasis and an atypical visceral form. Current treatments are hindered by toxicity, resistance, and high cost, driving the need for new therapeutic targets and drugs. N‐myristoyltransferase (NMT) is an important anti‐leishmanial target. N‐myristoyltransferase (NMT) is an important target in *Leishmania* parasites, as it plays a crucial role in the process of myristoylation, a lipid modification that involves the attachment of myristate, a 14‐carbon saturated fatty acid, to the N‐terminus of specific proteins. In this work, a shape‐based modeling approach was employed to identify potential NMT inhibitors in *Leishmania amazonensis*. Using a pyrazole sulphonamide as a reference ligand, a five‐feature shape‐based model was developed and validated. Virtual screening of the DIVERSet EXP and CL libraries (~1 million compounds) prioritized the top 500 ranked molecules per subset based on the TanimotoCombo score. Molecular docking studies identified the three highest‐ranking compounds from each subset based on ChemPLP scores and docking pose consistency. Among the selected ligands, CL 54016012, EXP 6689657, and EXP 9226834 exhibited the most favorable binding interactions, with CL 54016012 forming stable hydrogen bonds with Tyr80, Tyr217, and Tyr345. Molecular dynamics (MD) simulations indicated that ligand binding did not significantly alter NMT structural stability, although variations in binding energy and hydrogen bond were observed. CL 54016012 demonstrated the highest docking score, optimal RMSD stability, and the lowest predicted IC50 value (19.81 μM), suggesting its potential as a lead compound. In vitro cytotoxicity assays revealed that CL 54016012, CL 74995016, and EXP 6689657 reduced *L. amazonensis* viability in a dose‐dependent manner, placing them as promising candidates for further investigation in anti‐leishmanial drug development.

## Graphical abstract

Leishmaniasis is a significant health threat due to its various clinical forms and the challenges posed by current treatments. This study identifies three promising chemical compounds, CL 54016012, CL 74995016, and EXP 6689657, as potential inhibitors of N‐myristoyltransferase (NMT) in *Leishmania amazonensis*, offering new avenues for therapeutic development.

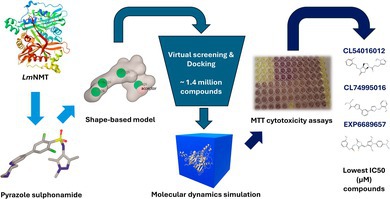

## 1 Introduction

Leishmaniasis is a neglected tropical disease caused by the protozoan parasites of the genus *Leishmania*, leading to significant morbidity and mortality worldwide. It is transmitted to humans by the bite of infected female sandflies. In Latin America, particularly in Brazil, leishmaniasis presents a significant public health challenge, with diverse clinical manifestations ranging from cutaneous to visceral forms (Saúde 2024). Among the species responsible for leishmaniasis in this region, *Leishmania amazonensis* is noteworthy due to its ability to cause both the anergic diffuse cutaneous form and the cutaneous forms characterized by disseminated lesions (Torres‐Guerrero et al. 2017). Moreover, *L. amazonensis* has been identified as a causative agent of atypical American visceral leishmaniasis, presenting with symptoms such as hepatitis and adenopathy, which further complicate the clinical diagnosis and management of this disease (Aleixo et al. 2006; Barral et al. 1986, 1991). The current leishmaniasis treatment options are limited by toxicity, resistance, and high cost, showing the urgent need for the discovery of new therapeutic targets and drugs with novel modes of action (Mendes Roatt et al. 2020).

Recent studies have highlighted the antiparasitic activity of natural terpenes from *Salvia cuspidata*, such as HABTO and ICTX, which exhibit promising in vitro and in vivo efficacy against *L. amazonensis* by inducing oxidative stress and mitochondrial dysfunction in the parasite (Troncoso et al. 2023). Immunotherapy, combining immunogenic mimotopes selected by phage display with the antifungal drug amphotericin B, has also shown a therapeutic response in mice infected with *L. amazonensis*, suggesting a potential for immunotherapeutics in managing the disease (Soyer et al. 2023). To date, several drug targets for leishmaniasis have been identified, such as P2X7 purinergic receptor (EL‐Dirany et al. 2022), eIF4A‐like protein (LieIF4A) (Abdelkrim et al. 2022), UDPase and PCNA (Carter et al. 2023), DNA topoisomerase (Roy 2022), bisubunit topoisomerase 1B (LdTop1) (Chowdhuri et al. 2023), and calcium‐transporting ATPases (Gupta et al. 2022) and others. One of such promising targets is the enzyme N‐myristoyltransferase (NMT), which catalyzes the attachment of myristate, a 14‐carbon saturated fatty acid, to the N‐terminus of specific proteins, a modification crucial for their function and association with cellular membranes (Brannigan et al. 2010). Frearson et al. (2010) identified a high‐affinity inhibitor (DDD85646) for *Trypanosoma brucei* that was indicated as an excellent chemical tool for the investigation of the biology of protein N‐myristoylation across a range of organisms. In this regard, recent research has focused on the inhibition of NMT as a strategy for the treatment of leishmaniasis. Structural studies have provided insights into the binding modes of inhibitors, aiding in the design of potent and selective NMT inhibitors (Brannigan et al. 2014). Besides, pharmacological validation of NMT as a drug target in *Leishmania donovani* has been achieved, with several potent inhibitors identified that demonstrate efficacy in reducing parasite burden in vivo (Corpas‐Lopez et al. 2019). The challenge remains to address the drop‐off in activity between enzyme inhibition and in vitro activity while maintaining selectivity over the human enzyme. Recently, novel thienopyrimidine inhibitors have shown on‐target activity in intracellular amastigotes of *Leishmania*, with excellent selectivity over human NMTs (Bell et al. 2020). Furthermore, natural products from *Withania somnifera* have been investigated as potential inhibitors of *Leishmania* NMT through molecular docking and dynamics studies, highlighting the role of natural compounds in drug discovery (Orabi et al. 2023).

The challenge in NMT‐inhibitor structural analysis lies in understanding the mechanisms by which the enzyme is inhibited. NMT can adopt open or closed conformations during its catalytic process. To address this, *in silico* methods have gained prominence. Among the computer‐aided drug design strategies that are shape‐based and pharmacophore models, highly effective tools in identifying inhibitors due to their ability to capture essential chemical features and spatial arrangements necessary for binding to a target enzyme or receptor. Pharmacophore models can be used in both ligand‐based and structure‐based approaches, allowing for the identification of diverse inhibitor binding modes. This versatility is demonstrated in studies targeting enzymes like chymase and PDE4, where multiple pharmacophore models were employed to capture different binding conformations and enhance screening efficiency (Arooj et al. 2013; Chen et al. 2010). Besides, a study conducted by shape‐based models effectively identified 32 compounds with 10%–38% inhibition at 10 μM against InhA reductase from mycobacteria (Kumar et al. 2013). Rather than relying solely on virtual screening, docking and on free binding energy calculations, research now focuses on modeling the conformational changes of the protein upon ligand binding. Molecular dynamics simulations play a crucial role in predicting inhibitor potency by assessing the conformational stability and dynamics of the protein in its closed form (Spassov et al. 2023a). Our work was based on the structure of the NMT‐inhibitor complex of *L. major* , which served as a model in the search for inhibitors by Frearson et al. (2010). This model was utilized to construct a ligand shape‐based model, subsequently employed for the search and identification of novel inhibitors using *in silico* tools and cytotoxicity assays. Leishmaniasis is an endemic and tropical disease that predominantly impacts low‐income regions (including parts of Africa, Asia, and Latin America) as well as middle‐income countries like Brazil. This study was conducted with the objective of advancing the quest for efficacious drug treatments against this disease.

## 2 Methods

### 2.1 Shape‐Based Modeling and Validation

To conduct a virtual screening against ligand libraries, we employed a shape‐based model. This model was constructed using color (pharmacophore features) and shape (inhibitor shape in the NMT pocket) based on the potent inhibitor, a pyrazole sulphonamide (DDD85646) complexed with *L. major* NMT (PDBid: 2WSA) available in the Protein Data Bank (PDB). The shape‐based model was created using the ROCS 3.2.0.4 program, OpenEye Scientific Software, Santa Fe, NM (Hawkins et al. 2007). To validate the shape‐based model, we employed the Receiver Operating Characteristic (ROC) curve and calculated the Area Under the Curve (AUC) using the ROCS 3.2.0.4 program. These metrics assess the performance of our models in distinguishing between active compounds (those binding to the target protein) and decoys (non‐target ligands or potentially inactive compounds). The active compounds were obtained from known ligands against the NMT protein, sourced from literature (Bell et al. 2012) and the inactives (decoys) were generated using the DUD‐E online platform (Mysinger et al. 2012).

### 2.2 Database Preparation

The shape‐based models generated through the ROCS program were employed in the virtual screening of compound libraries sourced from two databases: DIVERSet CL (core library) and DIVERSet EXP (express‐pick) from Chembridge Corporation, San Diego, California. CL contains approximately 870,000 diverse lead‐like compounds and EXP about 500,000 drug‐like structures carefully selected to cover a wide range of pharmacophoric space. The compound libraries were submitted to a two‐step processing: first, they were subjected to the FILTER program within OMEGA 2.5.1.4 (OpenEye Scientific Software, Santa Fe, NM) to eliminate undesirable compounds, including those with toxic properties. Next, the filtered databases were processed using the QUACPAC 1.6.3.1 program (OpenEye Scientific Software) to determine precise protonation states at pH 7.4 and generate tautomers for each molecule. Finally, the compounds were used to generate bioactive conformers through the OMEGA program.

### 2.3 Virtual Screening and Molecular Docking

The validate shape‐based model was employed for screening the DIVERSet subsets. Each screened library yielded 500 hits, representing the molecules with the highest overlap scores (referred to as TanimotoCombo). These top‐scoring 500 molecules, identified through shape‐based modeling, were subsequently subjected to molecular docking using PmNMT (PDBid: 2WSA) as the receptor. To accomplish this, we utilized the GOLD 2020.3.0 program (Jones et al. 1997). GOLD employs a genetic algorithm to optimize the ligand pose and orientation relative to the protein pocket. Gold ranks the best poses using the ChemPLP scoring function as default. 2D diagrams were generated using LigPlot+ to analyze the protein‐ligand interactions obtained from docking analyses (Wallace et al. 2021).

### 2.4 Molecular Dynamics

The three best scored ligands of each library from the molecular docking results were submitted to receptor‐ligand molecular dynamics (MD) simulations using the GROMACS software (Abraham et al. 2015). The topology parameters for each ligand were generated using SwissParam (Zoete et al. 2011) and the CHARMM force field. The MD simulations of the protein‐ligand complex were conducted in GROMACS, employing the CHARMM27 force field and the TIP3P water model. The simulation cell was defined as triclinic in shape. Water molecules and ions were added, and energy minimization was performed using the steepest descent algorithm. Both NVT (constant volume and temperature) and NPT (constant pressure and temperature) equilibrium phases were carried out over 100 ps, followed by a 100 ns production phase under NPT conditions. Particle mesh Ewald (PME) was employed to calculate long‐range electrostatic interactions during MD (Essmann et al. 1995). The temperature was maintained at a constant 300 K using a modified Berendsen thermostat (Berendsen et al. 1984), and the pressure was set to 1 atm using the Parrinello‐Rahman method (Parrinello and Rahman 1980). Short‐range van der Waals and electrostatic forces were set to 1.2. The MD results were evaluated using tools provided by the GROMACS program, including root‐mean‐square deviation (RMSD), root mean square fluctuation (RMSF) and hydrogen bond analysis.

### 2.5 Binding Free Energy Calculation

The binding free energy between the protein and the ligands was determined using the molecular mechanics Poisson–Boltzmann surface area method (MM‐PBSA) with energy decomposition, as proposed by (Massova and Kollman 2000). Specifically, we employed the gmx_MMPBSA tool for high‐throughput MM‐PBSA calculations (Valdés‐Tresanco et al. 2021). This approach allowed us to evaluate three key energy terms: vacuum potential energy, polar solvation energy, and non‐polar solvation energy. By conducting these analyses, we aimed to discern variations arising from different docked ligands.

### 2.6 Promastigote Culture

*L. amazonensis* (IFLA/BR/67/PH8) were cultured in Schneider medium supplemented with 10% FBS, penicillin (100 UI. mL^−1^) and streptomycin (100 μg. mL^−1^) at 23°C ± 0.5°C. Promastigotes used in all assays were isolated from the stationary growth phase.

### 2.7 Cytotoxicity Assays

Promastigotes (5 × 10^5^ cells/well) were placed on 96‐well culture plates and incubated at 23°C with different concentrations of the selected ligands (2‐fold serial dilution from 200 μM) for 48 h. The cell viabilities were evaluated by MTT assay according to Nunes et al. (2013) and the 50% inhibitory concentration (IC_50_) of ligands on cell viability was then determined by GraphPad Prism 5.0 (GraphPad Software Inc., San Diego, USA). The experiments were performed in quadruplicate and were conducted in at least two independent experiments.

### 2.8 Statistical Analysis

The statistical analysis was carried out using GraphPad Prism 5.0. For the determination of IC50 values, absorbance readings were plotted in a spreadsheet for the calculation of means and standard deviations. The values were normalized relative to the untreated control (considered as 100% viability). The tested concentrations were converted to logarithmic values for the construction of a nonlinear regression of the Sigmoidal dose–response type (variable slope) and for the determination of IC50 and 95% confidence interval (95% CI) values. Group comparisons were performed using one‐way ANOVA (analysis of variance) followed by Bonferroni's post‐test when appropriate. The results were evaluated at a statistical significance level of *p* < 0.05.

## 3 Results

### 3.1 Shape‐Based Model Validation and Screening for NMT Potential Ligands

**FIGURE 1:**
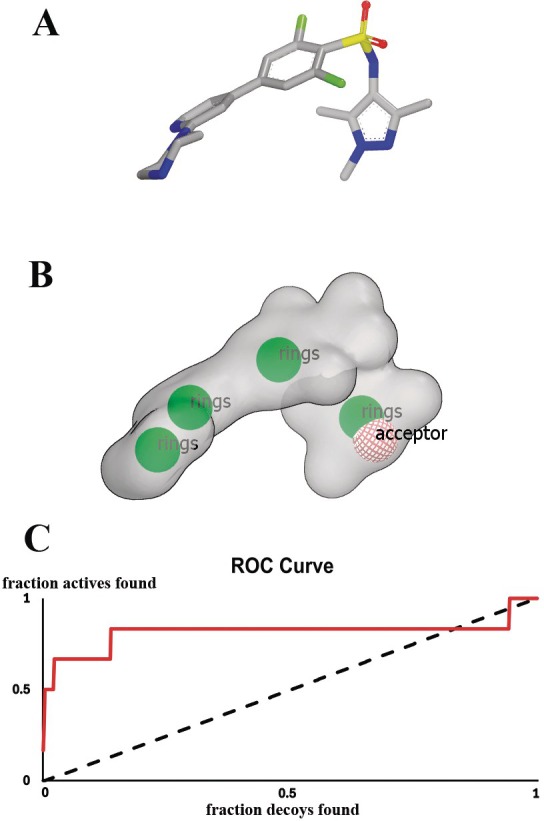
(A) DDD85646 inhibitor extracted from the complex with *L. major* NMT (PDBid: 2WSA). (B) Shape‐based model generated from DDD85646 pharmacophore features and shape. (C) Shape‐based model validation using ROC curve and AUC.

A shape‐based model approach was employed to select and screen potential inhibitors targeting NMT. The potent inhibitor DDD85646 complexed with *Leishmania major* NMT (*Lm*NMT), from the protein database (PDBid: 2WSA), served as the basis to construct a five‐pharmacophoric features shape‐based model (Figure 1A,B). Besides, a validation was conducted to identify the accuracy of the model in capturing potential active molecules (Figure 1C). The ascending curve denotes that the model exhibits a bias toward active molecules relative to inactive ones. Besides, the area under the curve (AUC) obtained for this model was 0.82, a good result, as indicated by ROCs manual; a highly selective query typically falls within the range of 0.8–1.0. The validated model was used to perform virtual screening using two subsets of molecules from the DIVERSet library (CL and EXP) and the outcome identified the top 500 ranked molecules from each subset, determined by the TanimotoCombo score.

### 3.2 Docking and Binding Mode Analysis

**FIGURE 2:**
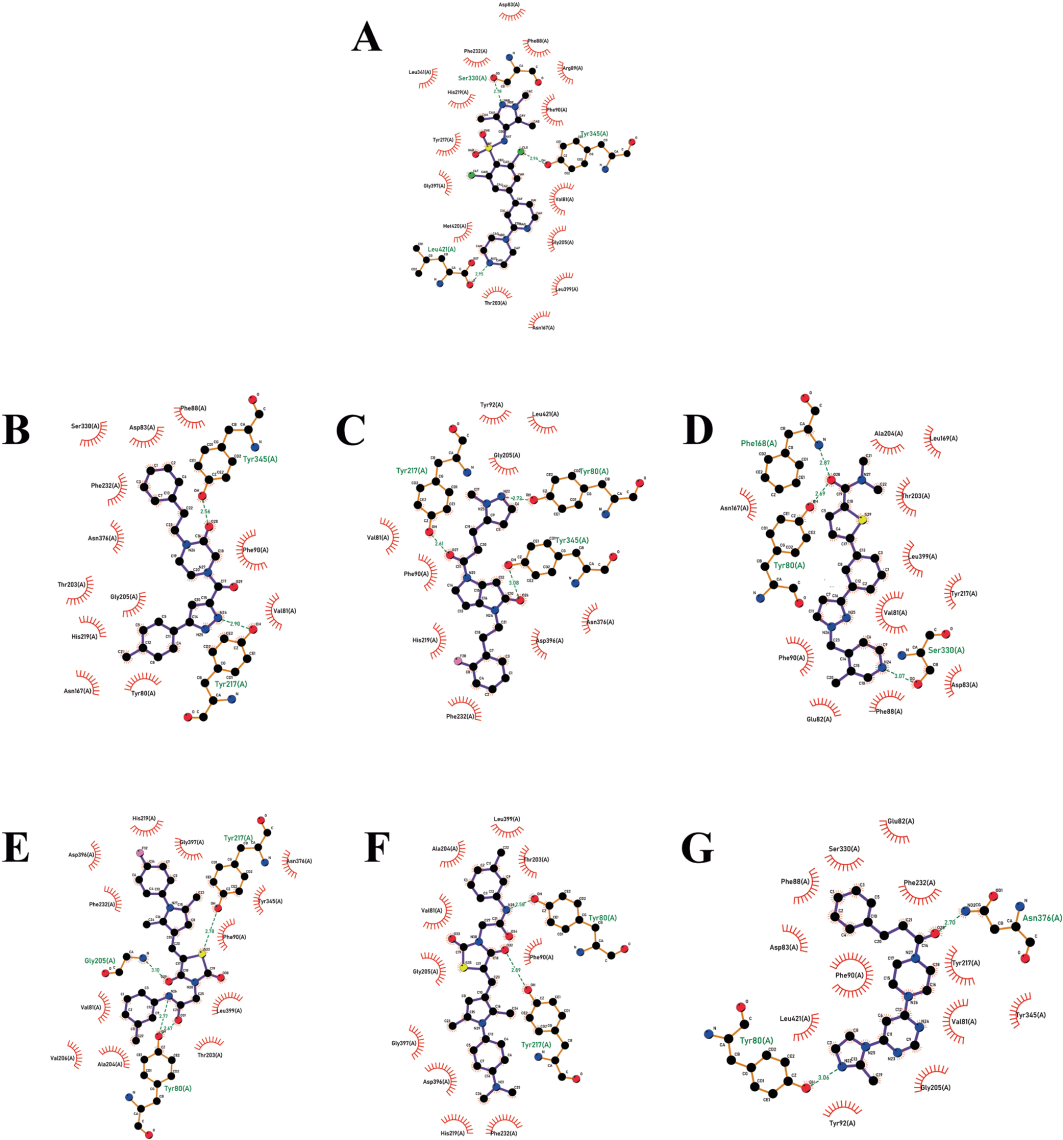
2D depiction of the DDD85646 inhibitor (A) and the best three ligands selected after docking, CL 19044540 (B), 54016012 (C), 74995016 (D) and EXP 6643498 (E), 6689657 (F), 9226834 (G). Spoked red arcs represent protein residues making hydrophobic contacts with the ligand and green dashed lines represents hydrogen bonds.

GOLD docking program was used to investigate the poses and the binding interaction between the *Leishmania major* NMT enzyme (LmNMT) and the top 500 ligands from each subset (CL and EXP), as determined by the Tanimoto Combo score. Additionally, the three best compounds were selected from each library based on the highest ChemPLP score (Table 1) and on converging to the same pose in the first three results from the 10 docking iterations of each molecule. It is important to note that despite very similar docking scores, ligands CL 54016012, EXP 6689657, and 9226834 showed the highest values, 86.48, 85.10, and 85.25 respectively. Subsequently, the selected ligands were evaluated for their interactions with LmNMT (Figure 2). DDD85646 performed 15 hydrophobic interactions and 3 hydrogen bonds (Ser330, Tyr345, and Leu421) with *LmNMT* (Frearson et al. 2010) (Figure 2A). CL ligands 19044540, 54016012, 74995016 (Figure 2B–D) performed 12, 9, and 11 hydrophobic interactions and 1 (Tyr345), 3 (Tyr80, Tyr217, and Tyr345) and 3 (Tyr80, Phe168, and Ser330) hydrogen bonds respectively. EXP ligands 6643498, 6689657, and 9226834 (Figure 2E–G) performed 12, 10, and 12 hydrophobic interactions and 3 (Tyr80, Gly205, and Tyr217), 2 (Tyr80 and Tyr217), and 2 (Tyr80 and Asn376) hydrogen bonds respectively.

**TABLE 1:** Virtual screening, docking, dynamics, and cytotoxicity results of the best docked ligands, apoenzyme, and DDD85646 inhibitor.

id	tanimoto combo (score)	chemplp (score)	rmsd (nm)	rmsf * (nm)	binding energy (kcal/mol)	h‐bond (number)	ic 50 (μm)
apoenzyme	—	—	0.18 ± 0.02	0.082 ± 0.031	—	—	
ddd85646	—	—	0.16 ± 0.02	0.076 ± 0.026	13.26 ± 8.48	1.77 ± 1.19	0.002**
cl							
19044540	1.23	82.60	0.15 ± 0.02	0.084 ± 0.030	−18.95 ± 5.02	0.36 ± 0.62	ND***
54016012	1.21	86.48	0.14 ± 0.01	0.084 ± 0.028	2.52 ± 6.53	2.31 ± 1.18	19.81 (13.98–29.42)^a^
74995016	1.20	81.47	0.22 ± 0.03	0.085 ± 0.029	25.64 ± 15.80	0.69 ± 0.70	29.30 (20.90–41.70)
exp							
6643498	1.29	82.34	0.16 ± 0.02	0.083 ± 0.028	−23.56 ± 4.02	0.06 ± 0.24	ND***
6689657	1.20	85.10	0.15 ± 0.01	0.083 ± 0.029	−19.93 ± 5.10	1.14 ± 0.72	101.90 (32.57–318.90)^a^
9226834	1.20	85.25	0.19 ± 0.03	0.088 ± 0.036	13.75 ± 6.41	0.01 ± 0.12	ND***

* Mean and standard deviation from the *Lm*NMT residues 343–421 (active site).

** Extracted from (Frearson et al. 2010) in vitro studies with *Trypanosoma brucei* NMT.

*** Not detected.

^a^ The comparative analysis of IC_50_ values (95% CI) showed that there was a statistically significant difference only between the compounds CL 54016012 and EXP 6689657.

### 3.3 NMT‐Ligand Complexes Stability Analysis

**FIGURE 3:**
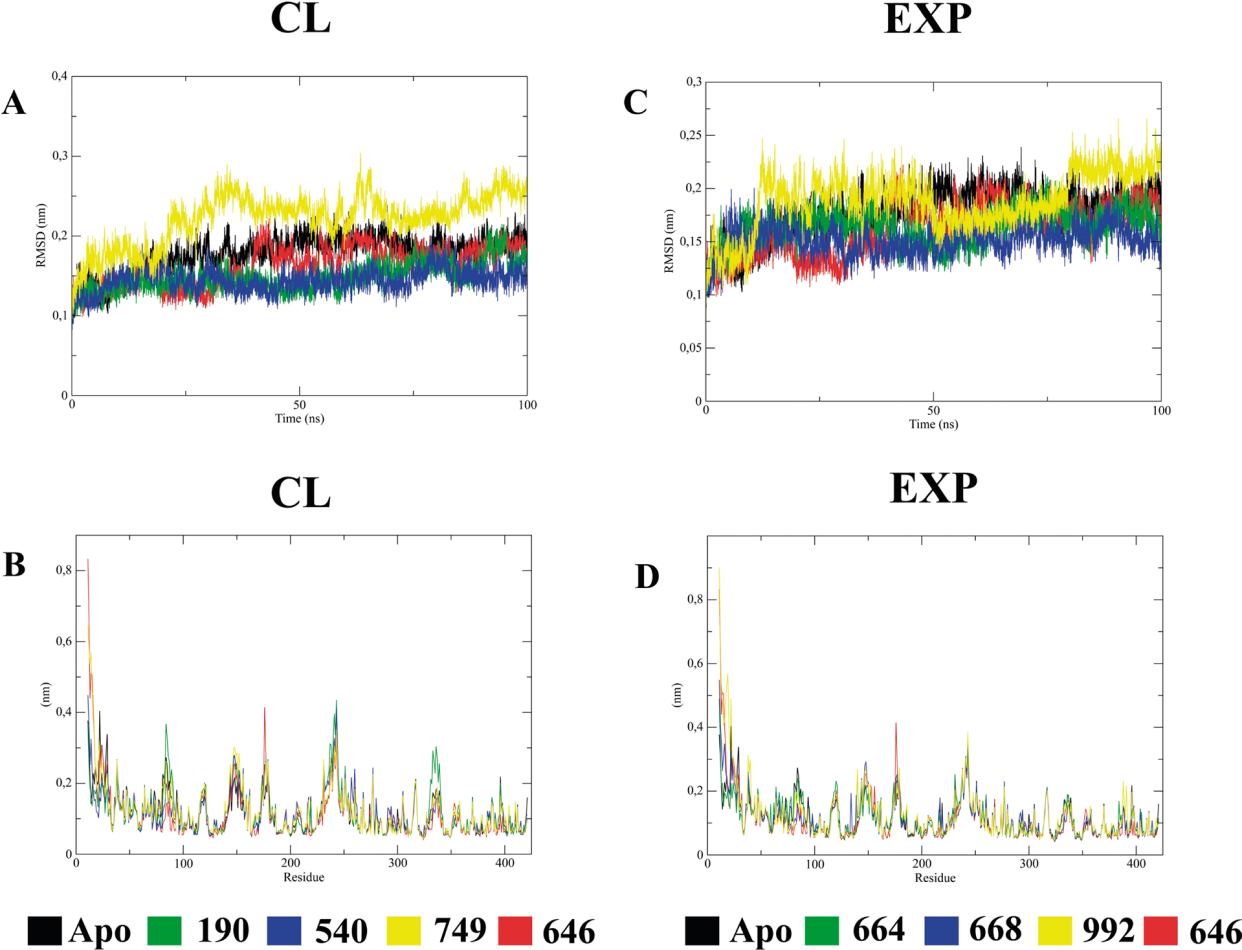
NMT‐ligand complexes and apoenzyme RMSD (A and C) and RMSF (B and D) analyses. NMT‐DDD85646 (646) and NMT apoenzyme (Apo) results are shown in all results. RMSD and RMSF results from NMT‐CL 19044540 (190), 54016012 (540) and 74995016 (749) complexes are shown in A and B. RMSD and RMSF results from NMT‐EXP 6643498 (664), 6689657 (668), and 9226834 (992) complexes are shown in C and D.

The RMSD and RMSF results from the 100 ns molecular dynamics of the NMT‐ligand complexes and the NMT apoenzyme were compared (Figure 3). All the CL (Figure 3A) and EXP (Figure 3C) NMT‐ligand RMSDs showed similar plateaus and means with the NMT‐DDD85646 and NMT apoenzyme over the 100 ns simulation except for the CL 74995016 and EXP 9226834, which showed slightly higher mean values (Table 1). Furthermore, it is worth noting that the NMT complexed CL 54016012 had the lowest RMSD mean and standard deviation.

The RMSF showed similar results among the complexes (Figure 3B,D) and apoenzyme. However, when the residues relative to the enzyme active site (residues 343–421) were analyzed (Table 1), we noticed that the NMT‐DDD85646 complex showed the lowest average fluctuation, probably due to the stabilization of the inhibitor interactions in the enzyme active site. Nevertheless, the NMT complex with EXP 9226834 showed the highest fluctuation (mean and standard deviation) of the residues in the enzyme active site (Table 1).

### 3.4 NMT‐Ligand Complexes Interaction Analysis

**FIGURE 4:**
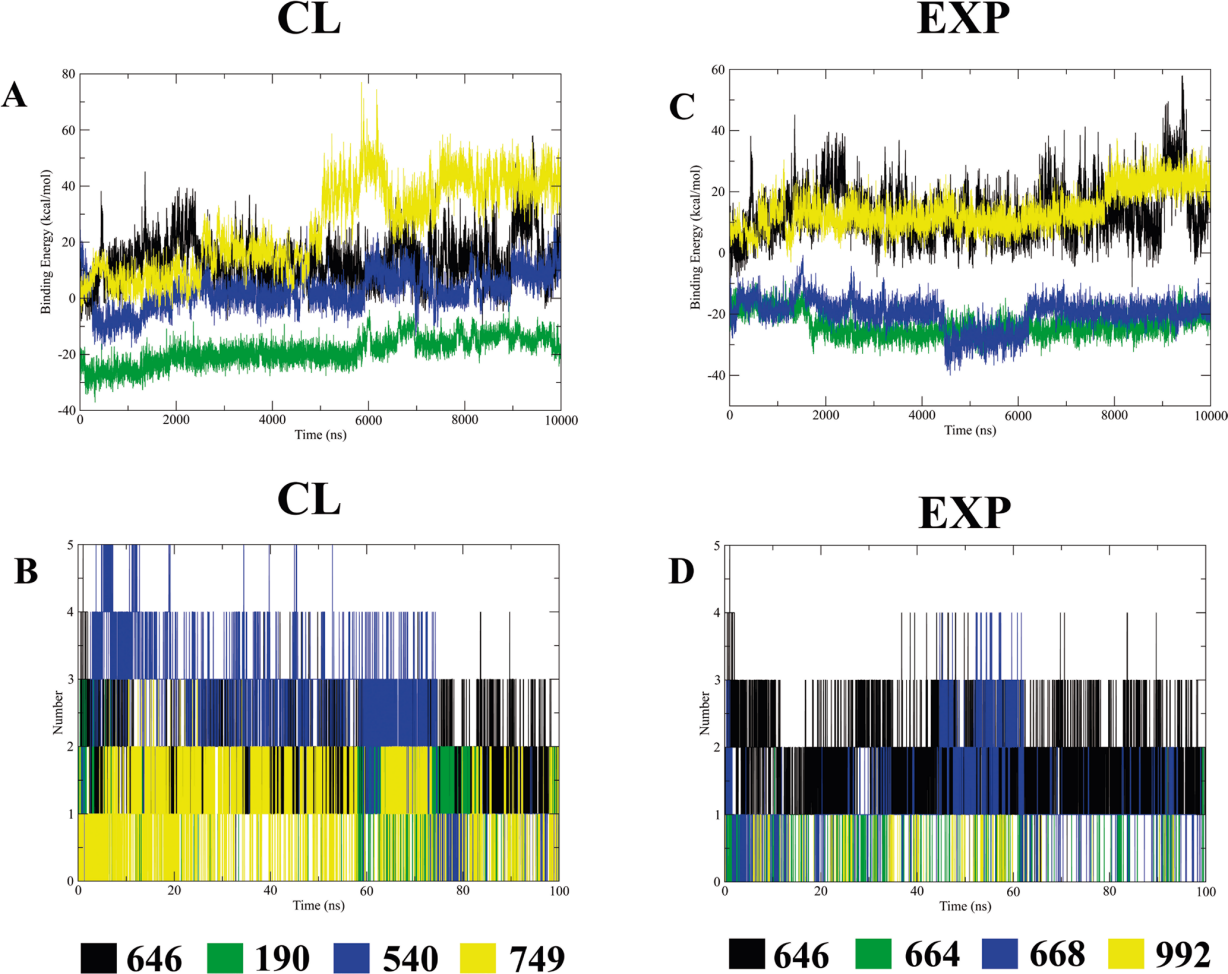
NMT‐ligand complexes binding energy (A and C) and hydrogen bond (B and D) analyses. NMT‐DDD85646 (646) are showed in all results. Binding energy and hydrogen bond results from NMT‐CL 19044540 (190), 54016012 (540), and 74995016 (749) complexes are shown in A and B. Binding energy and hydrogen bond results from NMT‐EXP 6643498 (664), 6689657 (668), and 9226834 (992) complexes are shown in C and D.

The investigation of the binding energy and hydrogen bond interactions during MD simulations provides valuable insights into ligand interaction stability within protein binding sites. Analysis over the 100 ns simulation period showed that the binding energy (kcal/mol) of the ligands in the site showed markedly different values along MD (Figure 4A,C). Notably, NMT‐DDD85646, CL 54016012, EXP 74995016, and 9226834 showed odd positive binding energy mean values, whereas NMT‐CL 19044540, EXP 6643498, and 6689657 showed similar negative mean values (Table 1). Regarding hydrogen bonds (Figure 4B,D) the complex NMT‐CL 54016012 showed the higher mean value, 2.31, quite similar to the 3 hydrogen bonds found in the initial complex generated by docking, despite having one of the worst binding energy mean values (2.52 kcal/mol) (Table 1). However, it is important to note that the hydrogen bonds of the complex fall off after 80 ns of simulation, which leads to a high standard deviation (1.18). Another interesting result is presented for the NMT‐EXP6643498 complex, which presented the lowest hydrogen bond mean (0.06) among the complexes studied, despite having the best binding energy mean value (−23.56 kcal/mol) (Table 1).

### 3.5 Potential Inhibitors Cytotoxicity

**FIGURE 5:**
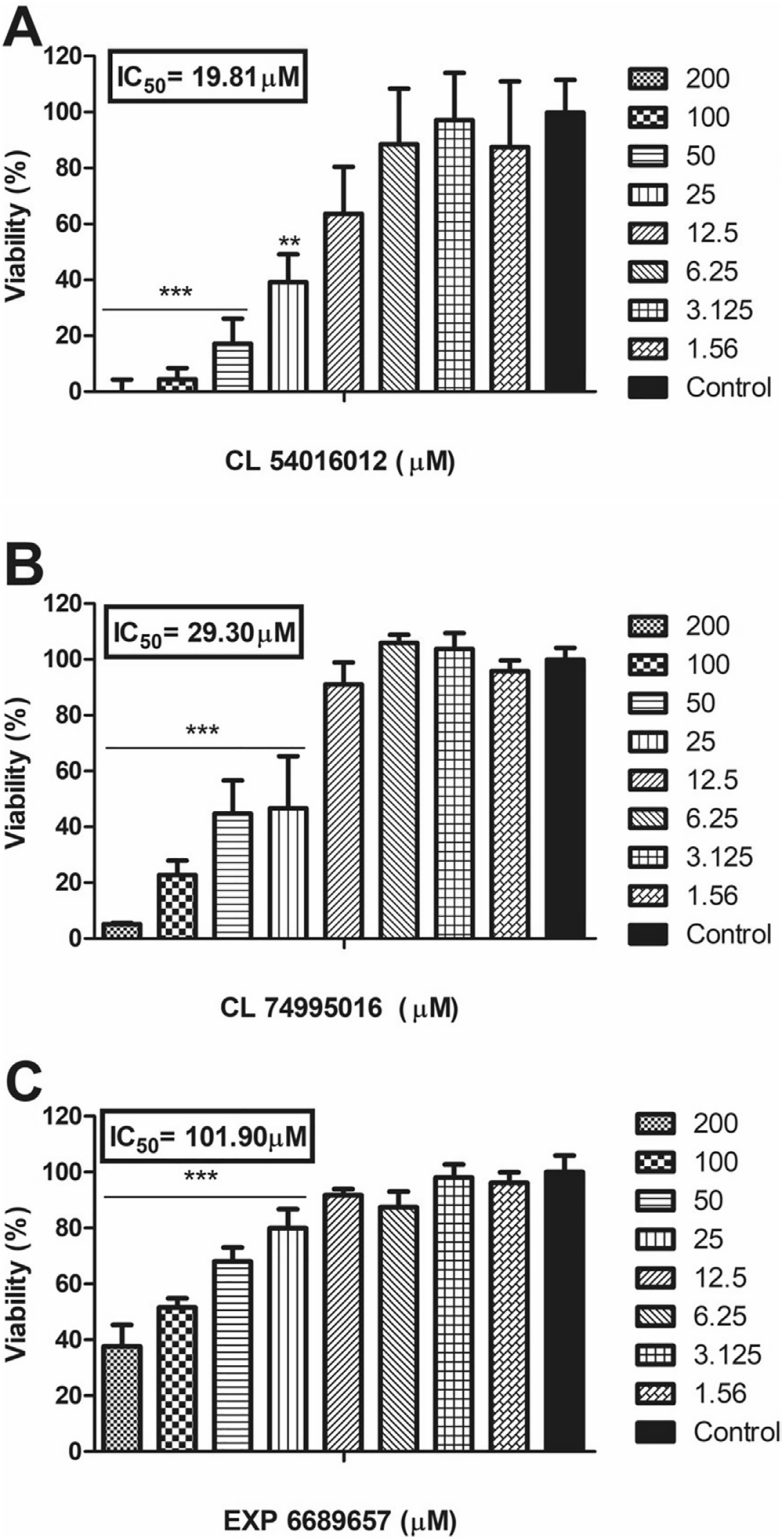
*Leishmania amazonensis* viability versus compounds CL 54016012 (A), CL 74995016 (B), and EXP 668965 (C). The viabilities of parasites were determined after 48 h in the presence of increasing doses of compounds. The graphs display cell viability percentages of parasites cultured with compounds relative to control parasites (100% of viability). Asterisks indicate statistically significant differences compared to the control (*** *p* < 0.001; **p < 0.01).

**FIGURE 6:**
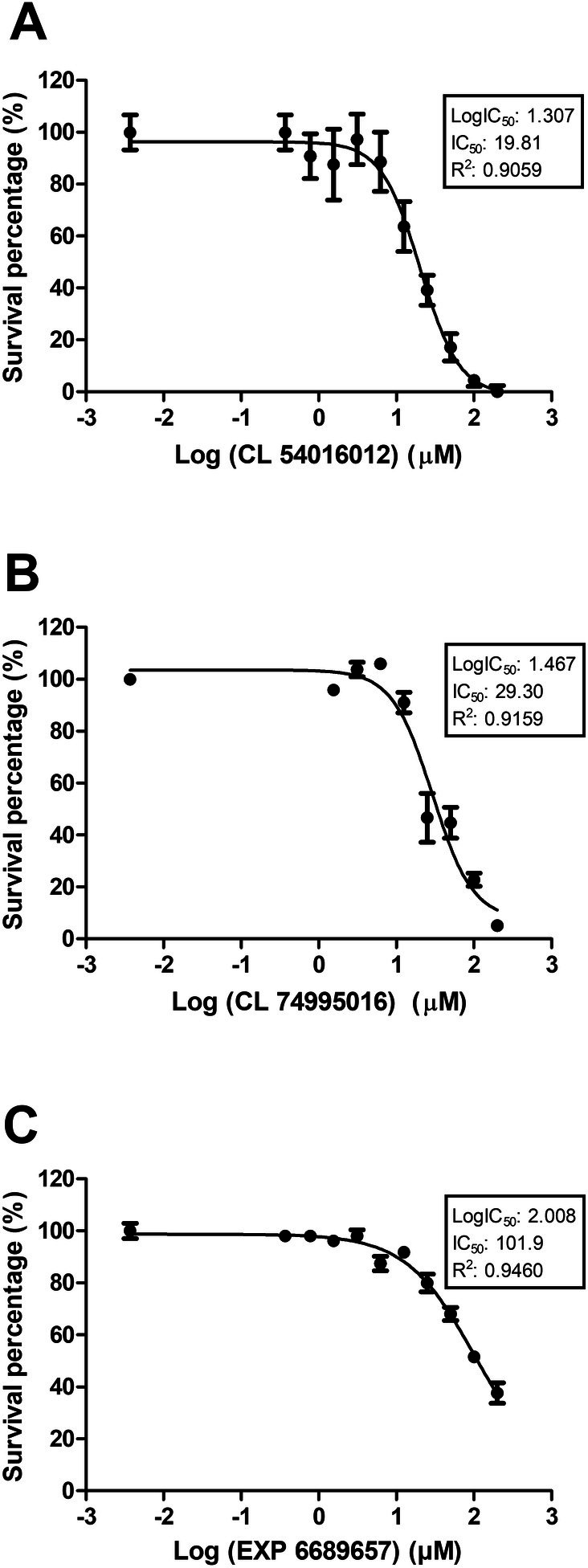
The log(concentration) versus percentage of survival graphs show the effect of the compounds CL 54016012 (A), CL 74995016 (B), and EXP 668965 (C) on the viability of *Leishmania amazonensis*. The graphs were generated from the data of the cytotoxicity assays and were used in the determination of the IC50 values using the GraphPad Prism software.

The six compounds (selected from the CL and EXP subsets) were acquired from ChemBridge Corporation, located in San Diego, California. These compounds were used in subsequent in vitro cytotoxicity assays against *L. amazonensis*. The results show that three compounds exhibited significant reductions in cell viability. The concentrations of compounds required to inhibit viability by 50% (IC_50_) were 19.81, 29.3, and 101.9 μM for CL 54016012, CL 74995016, and EXP 668965, respectively (Table 1). The graphs (Figures 5 and 6) depict the percentages of cell viability relative to control parasites (absence of compounds) versus compound concentrations and the log (concentration) versus percentage of survival. The dose–response profile analysis indicates concentration‐dependent viability inhibitions. However, the compounds CL 19044540, EXP 6643498, and 9226834 showed no detectable inhibition of viability in the cytotoxicity assays (Table 1).

## 4 Discussion

The pyrazole sulphonamide ligand (DDD85646) was used as a reference molecule in this work due to its prior findings on *L. major* binding site interaction and its potent effect in inhibiting *T. brucei* (Frearson et al. 2010). The shape‐based model constructed using DDD85646 was highly effective in selecting active compounds during validation. Thus, virtual screening was conducted to select from approximately 1 million molecules those that fit the ROCS shape‐based model better. The effectiveness of shape‐based models, particularly from ROCS, has been a topic of interest in the field of virtual screening and three‐dimensional shape retrieval. ROCS is a widely used tool for calculating 3D shape and chemical (“color”) similarity, which are crucial for identifying potential compounds in drug discovery (Kearnes and Pande 2016). The best 500 compounds from each subset (CL and EXP) were successfully docked to reveal the highest‐scoring poses in the *Lm*NMT binding site, guiding the subsequent steps in protein‐ligand MD. The importance of protein‐ligand MD simulations is widely acknowledged in the fields of computational biology and drug discovery. These simulations provide valuable insights into the dynamic nature of protein‐ligand interactions, which are crucial for understanding biological processes and designing new drugs. MD simulations offer a detailed view of how proteins interact with ligands in real time, allowing researchers to observe the conformational changes and dynamic behavior of proteins upon ligand binding (Karplus and Petsko 1990). This is particularly important because protein flexibility and conformational changes are often key factors in ligand affinity and specificity (Seo et al. 2014). The inhibition of NMT is a complex process that involves the stabilization of the enzyme in specific conformations. NMT can adopt open or closed conformations, which are crucial for its catalytic activity. A recent study indicates that the potency of NMT inhibitors is largely determined by their ability to stabilize the enzyme in its closed conformation. This stabilization effectively traps the inhibitor within the enzyme, creating a significant barrier to dissociation and enhancing the inhibitor's potency (Spassov et al. 2023a). Another key feature in the mechanism of NMT inhibition is the formation of salt bridges between the inhibitor and the enzyme. This interaction involves a positively charged chemical group of the inhibitor forming a salt bridge with the negatively charged C‐terminus of the enzyme, which significantly stabilizes the NMT‐ligand complex (Spassov et al. 2023b).

The MD results from NMT‐ligand complexes showed similar behavior in protein stability when RMSD and RMSF were analyzed indicating that ligand interaction presented a small impact on the behavior of NMT atoms (Figure 3 and Table 1). However, the results of the binding energy and the number of hydrogen bonds showed quite different results among the complexes (Figure 4, Table 1). The average binding energy results presented conflicting outcomes since some complexes displayed unexpectedly positive average values for the binding energy. This discrepancy may stem from the limitations of the MMPBSA technique used for these specific complexes, as the results are not consistent with the stability tests (RMSD and RMSF), the number of hydrogen bonds, and the in vitro findings. Regarding hydrogen bonding, NMT‐CL 54016012 exhibited the highest mean when analyzing the 100 ns of simulation. Additionally, this complex demonstrated the best RMSD stability, the highest docking score, and the lowest IC_50_ concentration in the toxicity tests (Table 1). It is important to note that hydrogen bonds play a pivotal role in molecular recognition and the binding affinity between proteins and ligands. These interactions are not static but dynamic, contributing to the flexibility and adaptability of protein‐ligand complexes (Bulusu and Desiraju 2020). Despite the observed stabilization of the NMT‐ligand complexes in closed conformations observed in our results, salt bridges between the ligands and the protein were not detected.

Recent advancements in the development of N‐myristoyltransferase (NMT) inhibitors have shown promising results in the fight against *Leishmania*. A study has demonstrated the structure‐based design of potent and selective inhibitors of *Leishmania* NMT, which were derived from a high‐throughput screening. These inhibitors showed a 40‐fold increase in enzyme inhibition through the hybridization of two distinct binding modes, resulting in novel compounds with significant potency against *Leishmania donovani* NMT and selectivity over the human enzyme (Hutton et al. 2014). Another research effort focused on a thienopyrimidine series, which was identified in a high‐throughput screen against *Leishmania* NMT. A comprehensive structure–activity relationship (SAR) study across 68 compounds led to the identification of the first inhibitor with on‐target NMT activity in *Leishmania* parasites. Crystal structure analyses of derivatives complexed with *Leishmania major* NMT revealed key factors for future optimization, resulting in a compound with modest activity against *L. donovani* intracellular amastigotes and excellent selectivity for *Leishmania* NMT over human NMTs (Bell et al. 2020). Pharmacological validation of NMT as a drug target in *L. donovani* was achieved by screening a focused set of pyrazolyl sulfonamide compounds. The most potent inhibitor showed modest activity against *L. donovani* intracellular amastigotes and a moderate therapeutic window over the human enzyme. This study confirmed the on‐target action of the inhibitor within parasites and demonstrated a significant reduction in parasite burden in an animal model of visceral leishmaniasis (Corpas‐López et al. 2018). Natural products have also been explored as potential NMT inhibitors. Compounds from *Withania somnifera* were subjected to molecular docking and dynamics against *L. major* NMT, identifying molecules with high affinity and binding stability, suggesting their potential use as antileishmanial drugs or as scaffolds for designing new inhibitors (Orabi et al. 2023).

It is important to notice that computational methods have become integral to drug design, offering significant advantages in terms of speed and cost‐effectiveness. However, these methods have limitations. One major issue is the accuracy of predictions. For instance, while computational models can predict drug‐target interactions, their accuracy is often limited by the quality and quantity of available data, as well as the inherent complexity of biological systems (Zhou 2019). Given these limitations, experimental validation remains crucial in drug design. Computational predictions need to be corroborated with experimental data to ensure their reliability and applicability.

To date, this is the first study to investigate potential inhibitors for the NMT of *Leishmania amazonensis* both *in silico* and in vitro. Our results are promising, highlighting three molecules, CL 54016012, CL 74995016, and EXP 668965, which could be used in future in vitro and in vivo studies to confirm their binding to NMT and their effectiveness in treating the disease. Furthermore, they could be further modified and refined to achieve better outcomes in future experiments.

## 5 Conclusion

The recent discovery of selective NMT inhibitors offers a promising avenue for the development of new antileishmanial therapies. This study screened 1 million compounds to identify prominent compounds against NMT from *Leishmania amazonensis*. Our results pointed to three potential NMT ligands that showed promising results in cytotoxicity assays, with CL 54016012 showing the lowest IC_50_ value. However, more tests need to be carried out to better understand their relationship with the NMT of *Leishmania* spp. and its human counterpart, enabling their potential use in in vivo tests and as a treatment alternative for this important and neglected disease.

## Conflicts of Interest

The authors declare no conflicts of interest.

## Data Availability

The data that support the findings of this study are openly available in Repositório Institucional (UFU) at https://repositorio.ufu.br/.
